# External Influence of Early Childhood Establishment of Gut Microbiota and Subsequent Health Implications

**DOI:** 10.3389/fped.2014.00109

**Published:** 2014-10-09

**Authors:** Peris Mumbi Munyaka, Ehsan Khafipour, Jean-Eric Ghia

**Affiliations:** ^1^Section of Gastroenterology, Department of Immunology and Internal Medicine, University of Manitoba, Winnipeg, MB, Canada; ^2^Department of Animal Science, University of Manitoba, Winnipeg, MB, Canada; ^3^Department of Medical Microbiology and Infectious Diseases, University of Manitoba, Winnipeg, MB, Canada; ^4^Inflammatory Bowel Disease Clinical and Research Centre, University of Manitoba, Winnipeg, MB, Canada

**Keywords:** antibiotics, cesarean section, diet, gut microbiota, immunity, inflammatory diseases

## Abstract

Postnatal maturation of immune regulation is largely driven by exposure to microbes. The gastrointestinal tract is the largest source of microbial exposure, as the human gut microbiome contains up to 10^14^ bacteria, which is 10 times the number of cells in the human body. Several studies in recent years have shown differences in the composition of the gut microbiota in children who are exposed to different conditions before, during, and early after birth. A number of maternal factors are responsible for the establishment and colonization of gut microbiota in infants, such as the conditions surrounding the prenatal period, time and mode of delivery, diet, mother’s age, BMI, smoking status, household milieu, socioeconomic status, breastfeeding and antibiotic use, as well as other environmental factors that have profound effects on the microbiota and on immunoregulation during early life. Early exposures impacting the intestinal microbiota are associated with the development of childhood diseases that may persist to adulthood such as asthma, allergic disorders (atopic dermatitis, rhinitis), chronic immune-mediated inflammatory diseases, type 1 diabetes, obesity, and eczema. This overview highlights some of the exposures during the pre- and postnatal time periods that are key in the colonization and development of the gastrointestinal microbiota of infants as well as some of the diseases or disorders that occur due to the pattern of initial gut colonization.

## Introduction

The human body houses trillions of microbes that are found in different parts of the body. The largest microbial population of the human microbiome is found in the gastrointestinal tract, and the greatest prevalence is in the colon, which is estimated to harbor 10^14^ bacterial cells and more than 100 times the number of genes in the human genome (1–3). The gut bacteria play an important role in human health by promoting intestinal homeostasis, stimulating the development and maturation of the immune system, protecting against pathogens, digesting fibrous food materials through fermentation, and harvesting nutrients (4–6). An alteration of the gut microbiota has been associated with an increasing number of diseases including inflammatory bowel disease (IBD), obesity, diabetes, asthma, and allergies (6, 7).

The current widespread use of high-throughput molecular microbiology techniques has enhanced our knowledge about the development of the intestinal microbiota to greater levels than were possible with classical culture techniques (8, 9). As a result of comprehensive microbiota analyses over time, insights into fundamental questions about the human microbiome dynamics under different microbial and host conditions are increasing (10). Longitudinal studies including the Human Microbiome Project have investigated the diversity of the bacterial population associated with the human body, its variability within and between individuals, the impact of internal and external factors, as well as characterizing its key components (8, 11, 12), and there is still much to learn. The human gut microbiota of a healthy adult is thought to be highly resilient and stable over time, a condition that may differ from one individual to another (13, 14). However, before reaching maturity, the gut microbiota needs to develop and establish a mutual beneficial co-existence with the host.

It has been suggested that the first contacts with pioneer bacteria could be deterministic for subsequent gut maturation, metabolic and immunologic programing, and consequently for short- and long-term health status (15). In addition, although there are discrepancies, metadata analysis over time has suggested an association between the nature of the initial gut microbiota colonization or microbial dysbiosis and a number of disease conditions in infancy and later in life (16, 17). This review will summarize some of the key factors (pre and postnatal) underlying the establishment of the neonatal gut microbiota and identify its potential impact on some of the immune-, intestinal-, and metabolic-related diseases in childhood or adulthood.

## Impact of the Prenatal Period on Gut Microbiota

The intrauterine environment and the unborn infant are generally thought to be sterile until delivery. However, some studies have reported the presence of bacteria in the intrauterine environment, which suggest that these bacteria may influence the microbiota of the infant before birth (18–22). For example, *Lactobacillus* and *Bifidobacterium* DNA were detected in the placenta of vaginally and cesarean section-delivered infants (23), and *Enterococcus faecium* strains that were orally inoculated to pregnant mice were later detected in the amniotic fluid and meconium of the pups following delivery (20). In addition, a unique placental microbiome niche similar to the human oral microbiome composed of non-pathogenic commensal microbiota from different phyla including Firmicutes, Tenericutes, Proteobacteria, Bacteroidetes, and Fusobacteria were characterized from a population-based cohort of placental specimens (24). This suggests that the placenta is not actually sterile and that oral microbiota may play a major role in the colonization of the placenta although the mechanism through which oral microbes find their way into the placenta remains to be elucidated. It is therefore possible that bacteria in the intrauterine environment could result in prenatal colonization of the meconium (6, 25). Jimenez and colleagues showed that the presence of bacterial species in the meconium, such as *Escherichia coli, E. faecium*, and *Staphylococcus epidermidis*, could result from the translocation of the mother’s gut bacteria via the bloodstream (19). Despite these findings, it is not clear whether colonization of the infant’s gut microbiota starts before birth, because the presence of bacteria in the amniotic fluid could also be an indication of undetected infection, which may increase the risk of miscarriages or preterm delivery (20). In this case, an association of the placental microbiome with a remote history of antenatal infections such as urinary tract infection in the first trimester and preterm birth has been reported (24). However, as reviewed by Li et al. (6), *Bifidobacterium* has been reported in meconium, amniotic fluid, fetal membranes, umbilical cord blood, and placenta of healthy mothers and infants with no detectable or known clinical infections or inflammation.

External factors during pregnancy may also influence the future development and behavior of the infant. For example, infant monkeys born from mothers stressed during pregnancy had significantly lower counts of *Bifidobacterium* and *Lactobacillus* (26). Probiotic administration (*Lactobacillus rhamnosus*) to mothers during late pregnancy also resulted in increased fecal *Bifidobacterium longum* counts in their infants (27), although, it is not clear whether these microbes were acquired from the mother during pregnancy, during birth, or after birth. Several other variables during pregnancy, including the use of antibiotics in the perinatal period, have been associated with delayed colonization by some microbes especially *Bifidobacteria* and *Lactobacillus* species (28, 29). This may have long-term impacts since these species are considered to have beneficial properties; for example, allergies, irritable bowel syndrome (IBS), and IBD, have all been frequently reported in antibiotic-exposed children (30–36). Roberts et al. (37) showed that children born to mothers who smoked have a higher risk of IBD, which may be due to disturbed microbial colonization since the cessation of smoking was correlated with increased Firmicutes and Actinobacteria, and a lower proportion of Bacteroidetes and Proteobacteria (38).

The length of the gestational period may also play an important role in initial infant gut microbial colonization. Colonization in preterm infants has been shown to take place slowly, have a low diversity, and several interindividual differences as opposed to that of full-term infants (6, 39). It is also mostly dominated by potential pathogens including *Clostridium* species, *E. coli, Enterococcus, Streptococcus, Klebsiella*, and *Staphylococcus* (6, 29, 39, 40). Healthy full-term infants are usually colonized by beneficial microbes, such as *Bifidobacterium* and *Lactobacillus*, which are not present or are detected in low levels in preterm infants (41). The delayed rate of colonization could result from the events surrounding the delivery, because most preterm infants are delivered through emergency or elective cesarean delivery. Infants born by cesarean section, notably electively, have been shown to have low bacterial richness and diversity (42), which could be a result of less exposure to the mothers’ delivery fluids, delayed oral feeding, and high hygienic care of the preterm infants and use of antibiotics that may consequently lead to colonization by few resistant/notorious microbes that are potentially pathogenic (6).

## External Factors Affecting the Development of Gut Microbiota in Infants During and after Birth

A number of factors (both intrinsic and extrinsic) may influence the process of microbial colonization in infants, which may consequently affect the infant’s health. The following section highlights some of the external factors that affect the initial colonization and establishment of gut microbiota in infants (Table 1).

**Table 1:** **Summary of the factors affecting gut microbiota colonization in infants**.

(a) Factors affecting colonization of gut microbiota before birth	(b) Factors affecting colonization of gut microbiota during/at birth	(c) Factors affecting colonization of gut microbiota after birth
- Intra-uterine environment - Maternal exposures	- Mode of delivery (caesarean section vs vaginal delivery)	- Breastfeeding vs formula feeding - Weaning or food
or practices such as stress, antibiotic use, smoking	- The environment at the time of delivery - Contact with the	supplementation - Antibiotic exposure
- Length of gestation period (term vs preterm)	mother or health care staff	
		- Home or family setting (rural vs urban)
		- Home structure (contact with the mother and other family members including siblings and close contact relatives
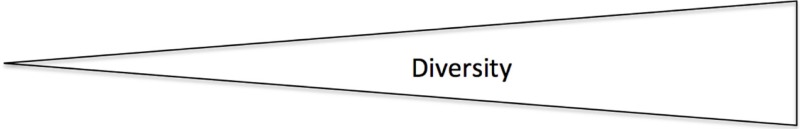		

### Delivery mode

During the birth process and immediately after birth, microbes from the mother and surrounding environment colonize the gastrointestinal tract of the infant leading to the development of a dense complex microbiota (22). The mode of delivery (vaginally or by cesarean section) has been demonstrated to have a strong influence on early gut colonization (43). A review by Mackie and colleagues (22) showed that, in cases of vaginal delivery, a longer birth process has been associated with the presence of viable microbes in the stomach and mouth of the infant, and the same *E. coli* serotypes were found in both the mouths of babies and in their mothers’ feces immediately after birth. This implies that the proximity of the birth canal and the anus play an important role in the transmission of microbes from the mother to the infant. In addition, bacteria present in the mother’s vagina immediately before birth were reported in the nasopharynxes of over 50% of babies born vaginally (22).

Children born by cesarean section are also exposed to their mothers’ microbiota, but initial exposure is most likely to non-maternally derived environmental isolates from equipment, air, and other infants, with the nursing staff serving as vectors for transfer (44). A number of studies have described altered fecal or intestinal microbiota profiles in cesarean section-delivered infants beginning at 1 day after birth and persisting to 6 weeks (7), 6 months, and even 7 years of age (45). Infants delivered by cesarean section have a lower total microbial diversity within the first 2 years of life associated with a less abundance and diversity of phylum Bacteroidetes (46). They are also less often colonized by *Bifidobacteria, Bacteroides*, and *E. coli*, but are more frequently colonized by both *Clostridium* cluster I and *Clostridium difficile* (46, 47). It has also been shown that skin microbes including *Staphylococcus, Corynebacterium*, and *Propionibacterium* dominate the gut microbiota of cesarean-delivered infants, while vaginally delivered infants have a higher prevalence of vaginal-related microbes such as *Lactobacillus, Prevotella*, and *Sneathia* (48–50). Generally, children born by cesarean section have an altered intestinal microbial colonization and studies have highlighted that this may be associated with a subsequent increased risk of developing various diseases including asthma and/or type 1 diabetes (T1D). This could be due to poor development of the immune system since infants born through cesarean section have also been reported to have remarkably lower levels of the Th1-related chemokines CXL10 and CXL11 in their blood, which may translate to less protection (46). A meta-analysis of the association between cesarean section and childhood asthma involving studies with different designs, conducted in different countries and using different measures of asthma reported an increased risk of asthma after cesarean section (17), whereas a 20% increase in the risk of childhood onset of T1D was reported in another meta-analysis of children born through cesarean section (51). An additional meta-analysis investigating the use of antibiotics in infants reported that the use of antibiotics in childhood was associated with asthma and wheezing (16).

### Diet

The succession of microbial colonization in the intestinal tract most occurs during the early development stages especially the first year of life. During this period, the feeding mode shifts from breastfeeding to formula feeding and/or to the introduction of solid food; however, individual instances of gut colonization may vary in terms of microbiota richness and diversity (7, 22, 52). Dynamic balances exist between the gastrointestinal microbiota, host physiology, and diet that directly influence the initial acquisition, developmental succession, and eventual stability of the gut ecosystem (53). Breastfeeding modulates the gut microbiota (54), and this might confer some protective effects to the infant against various forms of diseases or disorders (55), because evidence exists for an entero-mammary pathway that transfers diverse microbes from the mother’s gut to the baby through breast milk (56–59). However, human milk is known to contain complex polysaccharides that act as selective prebiotics and therefore promote the colonization of the infant gut with beneficial microbiota (60, 61), as opposed to children fed with formula. Formula feeding has been associated with an increased microbial richness of species in infants at four months of age with overrepresentation of *C. difficile*, a known gastrointestinal pathogen (42, 62). In addition, formula feeding induces intestinal hypertrophy and accelerates maturation of hydrolysis capacities; it increases intestinal permeability and bacterial translocation. Therefore, the microbiota may not be the principal actor. However, a recent publication observed more than two times increased numbers of bacteria cells in breast-fed infants, compared to formula-fed ones (63). It is therefore clear that breastfeeding may encourage proliferation or colonization by bacteria that may have protective effects on the growing infant, while formula feeding may predispose children to potential pathogens.

The gut microbiota of children is also influenced by the nature of food (other than formula) they receive, which could also be stratified by income status, mode of upbringing, or geographical location. The microbiota of children in Burkina Faso was found to be dominated by Bacteroidetes, compared with that of Italians, which was dominated by Firmicutes (64). Similarly, the biodiversity of microbiota from USA was lower than that from Malawians or Venezuelan Amerindians (65). Moreover, the effect of the natural environment, such as housing conditions, has also been investigated in animals. In this case, genetically related piglets were housed in either indoor or outdoor environments and sequencing of the 16S rRNA revealed that *Lactobacillus* strains were dominant in the gut of pigs raised outdoors, compared with hygienic indoor pigs, which had reduced *Lactobacillus* and more potentially pathogenic phylotypes (66).

### Mother and immediate family members

The influence of the mother on the child’s microbiota is evident during the first year after birth (43). This effect is reported to be stronger within the first month of life during which the infant’s intestinal microbiota is both functionally and phylogenetically close to that of the mother. However, at 1 year of age, phylogenetic differences appear while the similarities persist at the functional level (67). With respect to fecal microbiota, close similarities were found between the mother and the infant during the first six months after birth of the infant, which was mainly due to the presence of *Bifidobacterium bifidum, Bifidobacterium breve*, and *Staphylococcus aureus* (68).

After birth, environmental, oral, and skin microbes from the mother are mechanically transferred to the newborn in different ways, which may influence the diversity of intestinal microbiota in the neonate. For example, biodiversity in the homes, in the surrounding environment and in family members who have a close or constant contact with the baby have a direct impact on the diversity of microbes that are transferred to the infant (69–71). In addition, hygienic practices (e.g., cleaning of baby’s pacifier through sucking or by other methods) may influence the microbial diversity. Differences in microbial diversity have been associated with the development of allergy and/or asthma later in life (72, 73). Numerous population studies have also confirmed an inverse relationship between allergy prevalence and various measures of “hygiene,” such as growing up on a farm, early day care, and low socioeconomic standards (74, 75). There are also observed differences in the composition of the gut microbiota between infants living in countries with a high and a low prevalence of allergy and between healthy and allergic infants, even very early in life before they have developed any clinical symptoms of disease (76). In addition, an increasing number of older siblings are associated with the colonization of *Lactobacilli* and *Bacteroides* at 5 weeks of age, all of which are associated with beneficial effects (47). The mother and family at large, therefore, play a major role in the initial microbial colonization in infants who may have a health impact on the child. Table 1 shows a summary of the factors discussed in this review.

## Long-Term Effects of Microbial Colonization

Gut microbiota are generally associated with the development and maturation of the immune system (77). The immune system acquires most of its data from exposure to certain subsets of micro- and macro-organisms (78). For example, early colonization with *Escherichia coli* and *Bifidobacteria* is associated with higher numbers of CD20 + B cells that express the memory marker CD27 at 4 and 18 months of age (79). Disruption of exposure to these organisms is at least partly responsible for the immunoregulatory deficits that underlie the increased prevalence of conditions, such as chronic immune inflammatory diseases (i.e., IBD), asthma, and atopic dermatitis (7, 47, 80). Molecular microbiology techniques suggest that a high diversity of the gut microbiota in childhood could be more important as opposed to low diversity, which is associated with increased risk of subsequent allergic diseases, since repeated exposure to different bacterial antigens would enhance the development of immune regulation through inhibition of responses to inappropriate targets, such as gut contents and allergens (81, 82). The microbial diversity and composition of 47 infants as analyzed using barcoded 16S rRNA 454 pyrosequencing in stool samples at 1 week, 1 month, and 12 months of age, revealed that low total diversity of the gut microbiota during the first month of life was associated with asthma in the children at 7 years of age (83). A low gut microbial diversity during the first month of life was also associated with subsequent sensitization and atopic eczema at 2 years of age (82). In addition, reduced bacterial diversity of the infant’s intestinal microbiota was associated with increased risk of allergic sensitization, allergic rhinitis, and peripheral blood eosinophilia, in the first 6 years of life (81). Early-life exposures, including those known to impact gastrointestinal microbiome composition, such as antibiotic administration have also been associated with increased risk for childhood asthma due to altered microbiota profiles or long-term reduction in microbial diversity (7, 84, 85). Alterations of the intestinal microbiota in preterm infants characterized by low microbial diversity and abundance of potentially pathogenic bacteria have been highlighted in the development of necrotizing enterocolitis (NEC), although there are discrepancies among different studies (86, 87). Hällström and colleagues (88) reported a link between cesarean delivery and disturbed intestinal colonization, with an increased frequency of *Enterococcus* species and *Candida albicans*, and the probable occurrence of NEC in preterm infants. These findings may not be exclusively due to a disturbed intestinal microbiota because other confounding factors may also be responsible for NEC; however, further investigation will provide useful information on this topic and further clarify the existing discrepancies.

The birth order or family size and the presence or absence of pets have also been implicated in the initial microbial colonization in infants. Over two decades ago, Strachan observed that children who had older siblings were less likely to manifest hay fever as adults, as compared to firstborn children, which could be due to a protective effect of infections brought home by the older siblings (89). Similarly, exposure to livestock or pets, particularly dogs, early in life significantly decreases the risk for asthma and/or allergic reactions since dog ownership is associated with a distinct house dust microbial exposure (74, 90). In addition, mice exposed to dog-associated house dust were found to be protected against airway allergen challenge, because they exhibited less Th2 cytokine production, fewer activated T cells, and a distinct gut microbiome composition that was highly enriched for *Lactobacillus johnsonii*, which itself can confer airway protection when orally supplemented as a single species (91).

Selective microbial targets have been associated with infants developing eczema (92). Yap and colleagues (93) evaluated the composition of fecal microbiota of infants who developed eczema in the first 5 years of life compared with healthy controls and reported that longitudinal analysis of fecal microbiota composition at 3 days, 1 and 3 months, and 1 year of life showed a higher abundance of Enterobacteriaceae and *Clostridium perfringens* in children who developed eczema in the first 2 years of life, whereas a lower abundance of *Bifidobacterium* was observed in those who developed eczema at 5 years of age. The authors suggested that relative abundance of selective microbial targets might contribute to the subsequent development of eczema in childhood. Studies have also associated gut microbiota with the development of T1D (77). For example, metagenomic analysis revealed that the proportion of Bacteroidetes was increased in children with T1D while the proportion of Firmicutes was increased in normal healthy children (78). Conversely, the relative proportion of Bacteroidetes is decreased in obese people compared with lean people, while the proportion of Firmicutes is increased in obese people (78). In addition, colonization with clostridia, at the age of 5 and 13 weeks is associated with an increased risk of developing atopic dermatitis in the subsequent 6 months of life (47). Breastfeeding and formula feeding play major roles in defining the initial microbial colonization, and a previous meta-analysis showed that breast-fed children have a lower risk of being overweight compared to formula-fed children and that the duration of breastfeeding is inversely and linearly associated with the risk of overweight (94).

The use of antibiotics is generally known to change the gut microbiota. A meta-analysis on antibiotic use in infants reported that wheezing and asthma were related to antibiotic use (16). In addition, high-throughput sequencing revealed an incomplete, short-term recovery of infant gut microbiota following parenteral antibiotic treatment with ampicillin and gentamicin (95). The use of antibiotics in this context has been implicated in the development of IBD and current research has shown that children with IBD are more likely to have received antibiotics in their first year of life as compared to healthy controls (35), suggesting that microbial dysbiosis associated with early antibiotic exposure in neonates may be a predisposing factor to IBD, including other disease conditions, such as wheezing and asthma. Table 2 shows a summary of the factors affecting gut microbiota and their effects in neonates, infants or children, as discussed in this review.

**Table 2:** **Factors affecting colonization of gut microbiota in neonates and infants or children, specific microbial effect, and the resultant health conditions**.

Factor	Observed effect on microbiota	Specific health condition/disorder/disease	Reference
Intrauterine environment	Presence of bacteria in the uterus	Remote history of antenatal infections such as urinary tract infection during the first trimester Preterm birth	(18–25)
	Presence of bacteria in the amniotic fluid		
	Presence of bacteria in the meconium		
Stress during pregnancy	Low counts of beneficial bacteria (e.g., *Bifidobacteria, Lactobacillus*)	Allergic reactions	(26)
Probiotic use during pregnancy	Increased colonization by beneficial bacteria	Reduced incidence of allergic reactions	(27)
	Increased bacterial diversity		
Antibiotic use during pregnancy	Delayed colonization or reduced abundance of beneficial bacteria	Increased allergic reactions (asthma, allergic sensitization, allergic rhinitis)	(7, 28–36, 47, 80–83)
		Irritable bowel syndrome (IBS)	
		Inflammatory bowel disease (IBD)	
Smoking during pregnancy	Microbial dysbiosis (decrease in Firmicutes and Actinobacteria and an increase in Bacteroidetes and Proteobacteria	Increased risk of IBD	(37, 38)
Length of gestation period – preterm	Slow rate of bacterial colonization	Necrotic enterocolitis (NEC)	(6, 29, 39, 40, 86, 87)
	Reduced bacterial diversity		
	High interindividual differences in colonization		
	Increased level of potential pathogenic bacteria		
Length of gestation period – term	Increased abundance of beneficial bacteria	Lower incidence of NEC	(6, 29, 39–41, 86, 87)
	High bacterial diversity		
Cesarean delivery	Reduced bacterial richness and diversity	Increased risk of asthma, allergic reactions, Type 1 diabetes, atopic eczema, obesity and NEC	(6, 7, 17, 42, 46, 47, 51, 77, 78, 80, 88)
	Reduced colonization by beneficial bacteria		
	Increased colonization by potential pathogens		
		Low levels of Thl responses	
Vaginal delivery	Increased microbial diversity	Decreased risk of asthma, allergic	(48–50, 81, 82)

## Modifications of Early Childhood Microbiota

Several methods can be used to attempt to modify disturbed gut microbiota and many of them have been implicated in the improvement of gut microbiota in early childhood. However, an extensive discussion of the methods used to modify gut microbiota is beyond the scope of this review and only brief highlights on probiotics are included. As defined by the Food and Agriculture Organization/World Health Organization, probiotics are live microorganisms that, when administered in adequate amounts, confer health benefits on the host. The mechanisms by which probiotics exert beneficial effects on the host are currently a main area of focus although this may vary depending on the species or the strain involved. In this case, infants fed with a blend of *Lactobacillus acidophilus* and *Bifidobacterium infantis* as well as other probiotics had less cases of NEC and a reduced mortality rate compared to the controls (96), supporting the use of probiotics in preterm infants to prevent NEC. Although limited research has been done on the use of probiotics in NEC, probiotics appear promising for use as a prevention strategy for NEC; Abrahamsson and colleagues (97) argue that the time for confirmative NEC probiotic prevention trial in the extremely low birth weight infants in North America is now. On the other hand, the “hygiene hypothesis” suggests that a lack of exposure to microbial stimulus early in childhood is a major factor involved in the development of allergic reactions and immune-related disorders (89, 98), and therefore provides a rationale for using probiotics to modify the gut microbiota to shape the immune response of the host, especially in infancy. In this case, studies have provided evidence for a beneficial effect of different probiotics in the primary prevention and management of allergic diseases (atopic eczema, allergic rhinitis) (99, 100). For further reading on the use of probiotics in allergic diseases, the reader is referred to other detailed review articles (99–102).

## Summary and Conclusion

The characteristics of the inherited microbiome are likely very important in understanding offspring health as recent research suggests that aberrant metabolic phenotypes can be directly attributed to intestinal bacterial communities. Here, we have summarized some of the current interactions and associations on early-life exposures that may influence the development and colonization of the gut microbiota in infants and disease conditions that may result due to the nature of colonization. Some studies have already demonstrated the predictive power of the microbiota in enteric diseases while others have actually conducted research and consistently reported similar findings, although discrepancies do exist. In general, a reduction in overall bacterial diversity, a reduced abundance of commensal bacteria, and an increase in abundance of potentially pathogenic bacteria have been associated with immune-related disorders, and in some situations specific bacteria have being involved in the development of allergic reactions in both animal models and human studies. On the other hand, the importance of the gastrointestinal microbiome in defining the immune environment has been demonstrated in a number of studies, indicating that strategies to manipulate gut microbiome membership and function may have far-reaching implications for better health. This, therefore, shows that colonization by a certain subset of microbiota may be a threat or a benefit to the health of the infant during childhood and/or in adulthood. Consequently, future studies should focus on ways of promoting and/or maintaining colonization by the beneficial bacteria in infants who are exposed to compromising situations that encourage colonization by potentially pathogenic phylotypes. Prebiotics and/or probiotics have been suggested to have a promising impact in the prevention and treatment of some immune-related diseases by modulating gut microbiota and regulating host mucosal immune function; however, their efficacy is inconsistent and further studies and the exploration of other treatments are required.

Whether the altered microbiome causes the disease or is the disease affecting the microbiome remains an issue of debate. However, for neonates, it is possible to argue that the first colonizers play a major role in the development of the disease because if this is not the case, then why are some disorders/disease conditions more common in infants born via caesarian delivery compared with vaginally delivered infants? Or, why do formula-fed infants more frequently have metabolic disorders as opposed to breast-fed infants? Other questions include why is it that the majority of potentially known pathogenic microbes are found in children exposed to antibiotics, born by cesarean section, or raised under high hygienic conditions, whereas there are more known beneficial microbes in their counterparts? In support of our argument, differences in the composition of the gut microbiota between infants living in countries with a high and a low prevalence of allergy and between healthy and allergic infants, have been reported even very early in life before development of any clinical symptoms of disease (76), suggesting the role of the gut microbiota in the development of the disease conditions. This topic still remains open for discussion and the answers to the above questions will shed more light on this debate; future research should incorporate extended microbiota analyses, detailed nutrition assessments, and longitudinal measures of disease conditions throughout childhood.

Meta-analyses have substantiated the association between some disease conditions with cesarean section, formula feeding, and use of antibiotics in infants. However, these conditions may result from the impact of differences or changes already observed in the gut microbiota, or they could result from other confounding factors. Advanced molecular microbiology approaches including functional metagenomic analysis may shed more light on factors that affect infant microbial colonization and extend our knowledge on bacterial species that are potentially undesirable or actually beneficial, and that can be considered as potential probiotics for novel formula development in order to closely emulate nature’s models. Future studies should, therefore, focus on functionally characterizing the gut microbiota of children with the aim of identifying some of the metabolic pathways and processes that may be targeted for modulation with beneficial microbiota, and consequently aid in management of the disease conditions that are associated with altered gut microbiota or colonization by potential pathogens.

## Conflict of Interest Statement

The authors declare that the research was conducted in the absence of any commercial or financial relationships that could be construed as a potential conflict of interest.
